# Supplementary material for: Brain Injury Is Prevalent and Precedes Tobacco Use among Youth and Young Adults Experiencing Homelessness
Source: Int J Environ Res Public Health. 2023 Mar 15;20(6):5169. doi: 10.3390/ijerph20065169 (PMC10049052; doi:10.3390/ijerph20065169)
Supplement: Supplementary file 1 [file ijerph-20-05169-s001.zip › ijerph-2180432-supplementary.pdf]

International Journal of
Environmental Research
and Public Health

MDPI

SUPPLEMENTAL TABLES

Supplemental Table S1. Associations between the three brain oxygen deprivation exposure items and the composite blunt force head trauma measure; among the full sample with complete data.

	No BFHT (n=12)		BFHT (n=83)		
	n	*%*	*n*	*%*	*p-value*[1]
**Brain Oxygen Deprivation Exposure**					**<0.001**
None	11	91.67	22	26.83	
Any	1	8.33	60	73.17	
Choking Games					1.000
Never	12	100.00	72	86.75	
Once	0	0.00	5	6.02	
A few times	0	0.00	4	4.82	
Too many times to remember	0	0.00	2	2.41	
Intentional Choking					**0.009**
Never	12	100.00	39	46.99	
Once	0	0.00	12	14.46	
A few times	0	0.00	25	30.12	
Too many times to remember	0	0.00	7	8.43	
Stopped Breathing on Accident					0.161
Never	11	91.67	42	50.60	
Once	1	8.33	17	20.48	
A few times	0	0.00	17	20.48	
Too many times to remember	0	0.00	4	4.82	
Don’t Know	0	0.00	3	3.61	

[1] p-value from Fisher exact test

BFHT: blunt force head trauma exposure

Supplemental Table S2. Associations between the three blunt force head trauma exposure items and the composite brain injury oxygen deprivation measure; among the full sample with complete data

	No BOD (n=33)		BOD (n=61)		
	n	*%*	*n*	*%*	*p-value*[1]
**Blunt Force Head Trauma Exposure**					**<0.001**
None	11	33.33	1	1.64	
Any	22	66.67	60	98.36	
Hit in Head					**<0.001**
Never	20	60.61	9	14.75	
Once	2	6.06	8	13.11	
A few times	8	24.24	28	45.90	
Too many times to remember	3	9.09	16	26.23	
Shaken Violently					**<0.001**
Never	29	87.88	26	42.62	
Once	0	0.00	6	9.84	
A few times	4	12.12	15	24.59	
Too many times to remember	0	0.00	13	21.31	
Don’t Know	0	0.00	1	1.64	

	No BOD (n=33)		BOD (n=61)		
Accidentally Hurt					0.116
Never	15	45.45	13	21.31	
Once	6	18.18	14	22.95	
A few times	9	27.27	23	37.70	
Too many times to remember	3	9.09	11	18.03	

[1] p-value from Fisher exact test

BOD: brain oxygen deprivation exposure

Supplemental Table S3. Linear regression models for age first tried tobacco and age first regularly used tobacco among the full sample (N=96)

	Age Tried Tobacco			
	Unadjusted		Adjusted[a]	
	Years (95% CI)	p-value	Years (95% CI)	p-value
Brain Oxygen Deprivation Exposure vs. None	-1.15 (-2.69, 0.39)	0.141	-0.87 (-2.49, 0.76)	0.292
Blunt Force Head Trauma Exposure vs. None	-0.16 (-2.39, 2.07)	0.885	1.72 (-0.62, 4.06)	0.148
Brain Oxygen Deprivation or Blunt Force Hea Trauma Exposure vs. None	-0.09 (-2.41, 2.22)	0.937	1.84 (-0.63, 4.31)	0.142
Type of Exposure		0.243		0.086
None	0.51 (-1.84, 2.85)		-1.15 (-3.65, 1.35)	
Single Type	1.49 (-0.26, 3.25)		1.45 (-0.25, 3.16)	
Both	Reference		Reference	
Intentional Brain Oxygen Deprivation Exposur vs. None	-0.43 (-1.94, 1.08)	0.575	-0.42 (-1.94, 1.11)	0.586
Intentional Blunt Force Head Trauma Exposur vs. None	-0.89 (-2.61, 0.83)	0.307	-0.10 (-1.99, 1.80)	0.920
Intentional Brain Oxygen Deprivation or Blur Force Head Trauma Exposure vs. None	-1.24 (-2.98, 0.51)	0.163	-0.39 (-2.28, 1.50)	0.683
Type of Intentional Exposure		0.318		0.927
None	1.04 (-0.84, 2.92)		0.40 (-1.65, 2.45)	
Single Type	-0.56 (-2.39, 1.27)		0.10 (-1.69, 1.89)	
Both	Reference		Reference	
	**Age First Regularly Used Tobacco**			
	**Unadjusted**		**Adjusted**	
	**Years (95% CI)**	**p-value**	**Years (95% CI)**	**p-value**
Brain Oxygen Deprivation Exposure vs. None	-1.42 (-2.42, -0.41)	**0.006**	-0.87 (-1.95, 0.21)	0.113
Blunt Force Head Trauma Exposure vs. None	0.16 (-1.34, 1.66)	0.830	0.94 (-0.63, 2.51)	0.235
Brain Oxygen Deprivation or Blunt Force Hea Trauma Exposure vs. None	0.09 (-1.46, 1.65)	0.907	0.81 (-0.85, 2.47)	0.335

Type of Exposure		**0.008**		0.114
None	0.41 (-1.11, 1.93)		-0.30 (-1.99, 1.38)	
Single Type	1.81 (0.67, 2.95)		1.09 (-0.06, 2.23)	
Both	Reference		Reference	
Intentional Brain Oxygen Deprivation Exposur vs. None	-0.55 (-1.56, 0.45)	0.276	-0.47 (-1.48, 0.54)	0.359
Intentional Blunt Force Head Trauma Exposur vs. None	-0.92 (-2.05, 0.21)	0.108	-0.05 (-0.31, 1.21)	0.938
Intentional Brain Oxygen Deprivation or Blur Force Head Trauma Exposure vs. None	-0.95 (-2.11, 0.21)	0.108	-0.08 (-1.34, 1.18)	0.904
Type of Intentional Injury		0.228		0.615
None	1.06 (-0.17, 2.30)		0.36 (-0.99, 1.71)	
Single Type	0.19 (-1.02, 1.39)		0.57 (-0.61, 1.75)	
Both	Reference		Reference	

[a]Adjusted for race and the Behavioral Risk Factor Surveillance System Adverse Childhood Experiences Scale
